# Association between Dietary Patterns during Pregnancy and Birth Size Measures in a Diverse Population in Southern US

**DOI:** 10.3390/nu7021318

**Published:** 2015-02-16

**Authors:** Uriyoán Colón-Ramos, Susan B. Racette, Jody Ganiban, Thuy G. Nguyen, Mehmet Kocak, Kecia N. Carroll, Eszter Völgyi, Frances A. Tylavsky

**Affiliations:** 1 Department of Global Health, Milken Institute School of Public Health, George Washington University, 950 New Hampshire Avenue, Washington, DC 20037, USA; 2 Program in Physical Therapy, Department of Medicine, and Institute for Public Health, Washington University School of Medicine, 4444 Forest Park Avenue, Campus Box 8502, St. Louis, MO 63108, USA; E-Mail: racettes@wustl.edu; 3 Clinical and Developmental Psychology, Department of Psychology, Columbian College of Arts and Sciences, George Washington University, Room 304 Building GG 2125 St. NW, Washington, DC 20052, USA; E-Mail: ganiban@gwu.edu; 4 Department of Preventive Medicine, University of Tennessee Health Science Center, 600 Jefferson St. Room 337 Memphis, TN 38105, USA; E-Mails: tnguye41@uthsc.edu (T.G.N.); ftylavsky@uthsc.edu (F.A.T.); 5 Division of Biostatistics and Epidemiology, Department of Preventive Medicine, College of Medicine, University of Tennessee Health Science Center, 66 N. Pauline Street, Suite-633, Office 619, Memphis, TN 38105, USA; E-Mail: mkocak1@uthsc.edu; 6 Division of General Pediatrics, Department of Pediatrics, Vanderbilt University School of Medicine, 313 Oxford House, Nashville, TN 37232-4313, USA; E-Mail: kecia.carroll@vanderbilt.edu; 7 Department of Pediatrics (primary), Department of Preventive Medicine, University of Tennessee Health Science Center, Le Bonheur Children’s Hospital, Research Building, 50 North Dunlap Street, Room 477R, Memphis, TN 38103, USA; E-Mail: evoelgyi@uthsc.edu

**Keywords:** nutrition, pregnancy, birth weight, birth length, birth head circumference, African American, diet patterns

## Abstract

Despite increased interest in promoting nutrition during pregnancy, the association between maternal dietary patterns and birth outcomes has been equivocal. We examined maternal dietary patterns during pregnancy as a determinant of offspring’s birth weight-for-length (WLZ), weight-for-age (WAZ), length-for-age (LAZ), and head circumference (HCZ) Z-scores in Southern United States (*n* = 1151). Maternal diet during pregnancy was assessed by seven dietary patterns. Multivariable linear regression models described the association of WLZ, WAZ, LAZ, and HCZ with diet patterns controlling for other maternal and child characteristics. In bivariate analyses, WAZ and HCZ were significantly lower for processed and processed-Southern compared to healthy dietary patterns, whereas LAZ was significantly higher for these patterns. In the multivariate models, mothers who consumed a healthy-processed dietary pattern had children with significantly higher HCZ compared to the ones who consumed a healthy dietary pattern (HCZ β: 0.36; *p* = 0.019). No other dietary pattern was significantly associated with any of the birth outcomes. Instead, the major outcome determinants were: African American race, pre-pregnancy BMI, and gestational weight gain. These findings justify further investigation about socio-environmental and genetic factors related to race and birth outcomes in this population.

## 1. Introduction

Pregnancy is a critical period for the offspring’s metabolic development [1]. Inadequate maternal nutrient or energy intake during pregnancy is thought to lead to low birth size [2,3,4], a risk factor for infant and child mortality and morbidity, and potential risk factor for predisposition to cardiometabolic diseases later in life [5,6,7,8]. In the context of an increasingly energy-dense, nutrient-poor food environment in the US [9,10,11], there is increased interest in the promotion of nutrient-rich diets during pregnancy, with emphasis on iron-rich foods, folic acid, calcium-rich foods, and plenty of fruits and vegetables [12,13]. Dietary patterns are a way to capture the quality of the entire diet consumed by study populations.

Dietary patterns integrate dietary behaviors of a population through food and nutrient-group analyses. They are therefore more intuitive to public health nutrition recommendations than analyses that focus on single nutrients. Dietary patterns consider beneficial or harmful interactions among nutrients in different foods consumed together, as well as different food sources of the same nutrient [14]. The two most common approaches to study dietary patterns are *a priori* and *a posteriori* approaches [15]. The first one establishes *a priori* scores of foods and nutrients based on a hypothesis (e.g., adherence to the Mediterranean Diet Score [16], the Healthy Eating Index [17,18] or a score for junk food intake [19]). The second approach is exploratory, usually employing principal component analysis or factor analysis to generate patterns that maximally explain the variance in food intake in a population, where the results are data-driven and context-specific. Both approaches have been shown to be biologically meaningful [20,21]. For example, patterns characterized by a high intake of nutrient-poor, highly refined foods containing added sugar or unhealthy fats have been associated with biomarkers of inflammation and increased risk factors for cardiovascular disease, type 2 diabetes, and obesity compared to patterns characterized by high intake of lean proteins, vegetables, fruits and whole-grain cereals [15,22,23,24,25,26,27,28,29].

Few studies have examined the association between pregnancy or preconception dietary patterns and birth outcomes; most used principal component analysis or factor analysis [21,30,31,32,33,34,35] and approaches such as *a priori* scores [16,17,18,19,36,37,38]. In general, these studies suggest that energy-dense, nutrient-poor dietary patterns characterized by foods high in saturated and trans fats, refined sugar, or sodium are negatively associated with birth size outcomes [30,31,34,35,39], and that patterns characterized with nutrient-rich foods such as fruits, vegetables, and whole grains, were positively associated with birth size outcomes [30,31,33,34,35,39]. However, there are some inconsistencies in findings, probably due to the variation in birth size outcome measures, context of the population and resulting dietary patterns [31,32,39]. The majority of these studies have been conducted among white European or European American populations and, to date, no study has examined the association between maternal dietary patterns and birth size outcomes in a population with a high burden of low birth weight.

Due to the important differences in eating patterns by geography, culture and other context-specific characteristics of the population, we sought to determine the influence of specific dietary patterns on birth size outcomes in a diverse, largely black African-American and low-income population residing in the South of the US. The objective of this study was to examine the extent to which maternal dietary patterns are associated with offspring size at birth (birth weight, length, and head circumference). Dietary patterns were used to describe patterns that emerge from the data and display the unique features of that population which may not be captured by any predefined score. We hypothesized that dietary patterns characterized by energy-dense, nutrient-poor processed foods that are high in saturated and trans fats, sodium, and refined sugars would be associated with lower birth weight, length, and head circumference compared to healthy dietary patterns during pregnancy.

## 2. Materials and Methods

This analysis was conducted in a pregnancy cohort of 1151 women who were followed from the second trimester of pregnancy until delivery.

### 2.1. Setting

The Conditions Affecting Neurocognitive Development and Learning in Early Childhood (CANDLE) study is a longitudinal cohort study set in Shelby County, the southwestern corner of the state of Tennessee. Shelby County is largely African-American with mid-level education, and low income status (<200% poverty level). The CANDLE study aims to investigate the effects of different exposures such as mother’s prenatal habits and characteristics, home environment and childhood experiences, genetics, and exposure to potentially harmful substances on the neurocognitive development of children from birth to age three years. The study was conducted in accordance with the Helsinki Declaration and was approved and reviewed by the Institutional Review Board of the University of Tennessee Health Science Center on 17 June 2014 (approval code: 06-08495-FB).

### 2.2. Participants

Women were eligible to enroll in the CANDLE study if they were between 16–28 weeks pregnant, were a resident of Shelby County, had a low medical risk pregnancy, were between the ages of 16–40 years, spoke and understood English, had a single pregnancy and were willing to give consent. The CANDLE study recruited a total of 1503 pregnant women; 1474 mother-child dyads were available for follow up after excluding post-consent ineligibilities, pre-delivery withdrawals, and fetal demises. Of the 1474 participants, 1151 had diet data at the visit between 16–26 weeks, when their diet was assessed.

### 2.3. Variables

Outcomes were Z-scores for weight-for-length (WLZ), weight-for-age (WAZ), length-for-age (LAZ), and head circumference (HCZ). Exposure of interest was the maternal dietary patterns that have been previously assessed via factor analysis [40]. Independent variables were socio-demographic, behavioral, and medical history characteristics that would be considered as confounding variables in the association between pregnancy outcomes and diet.

### 2.4. Data Sources

Data used for this study were collected during the second trimester and at birth. During the second trimester, participants completed questionnaires asking about demographics, health status, diet, and medical history. At birth, research assistants conducted medical chart abstractions for birth outcomes (weight, length, head circumference).

*Diet Instrument*: Diet was assessed at enrollment (16–26 weeks of pregnancy) using the Block 2005 food frequency questionnaire (FFQ) that asks consumption of 111 food and drink items during the previous three months [41,42,43]. The Block FFQ has been shown to be a valid and reliable instrument to rank individuals according to dietary and nutrient intake [44]. Interviewers were trained by registered dietitians and re-certified by a registered dietitian based on a taped interview every six months to estimate the frequency and quantity of intake. Nutrient values were obtained from NutritionQuest (Berkeley, CA, USA). Over and under-reporters of total caloric intake (>5000 kcal per day or <1000 kcal per day) were excluded (*n* = 152).

*Dietary patterns:* Seven dietary patterns were identified previously using exploratory factor analysis with principal component extraction and varimax rotation method to determine the frequency of 111 food and beverage groups that made up distinct dietary patterns. Volgyi *et al.* [40] describe these patterns as: healthy (characterized by high factor loadings of vegetables, fruits, non-fried fish and chicken, and water); processed (*i.e.*, processed meat, fast food items, snacks, sweets, and soft drinks); Southern (*i.e.*, cooked cereals, peaches, corn, fried fish, beans, greens, pig’s feet, neck bones oxtails, tongue, pork); healthy-processed; healthy-Southern; Southern-processed, and mixed. The “mixed” pattern reflects foods from all of the other patterns together. In brief, to create the patterns, Volgyi and colleagues [40] estimated a factor score for each participant as a sum of daily frequency of intake of each food group, multiplied by the loading score for the food group. A large segment of the population belonged to mixed patterns rather than to single pure patterns (such as healthy, processed or Southern), so they then created combined food patterns based on the individual’s rank order in each single factor. Dietary patterns were assigned based on the individuals’ scoring in the quintiles for each food factor. These dietary patterns are distinct from each other in their content of energy-adjusted nutrients and explain more than 80% of the variance in macronutrient intake for this study population [40].

*The demographic survey*: Administered during enrollment asked respondents about formal education, medical insurance, annual household income, age, race and ethnicity.

*Maternal baseline data form*: At the time of enrollment, researchers collected the participant’s self-reported pre-pregnancy length and weight, tobacco use, alcohol use, and total number of pregnancies (including abortions, miscarriages, stillbirths and current pregnancy). Pre-pregnancy body mass index (BMI) was calculated using the self-reported heights and weights.

*Labor and delivery forms and neonatal summary forms*: At the time of labor and delivery, the following information was abstracted from the medical charts: maternal weight and newborn birth weight, length, and head circumference.

### 2.5. Data Analyses

For full term infants (≥37 weeks), WHO Child Growth Standards were used to calculate Z-scores for each outcome WLZ, WAZ, LAZ and HCZ [45]. Normal distribution of scores was assessed via Q-Q plots of residuals for each birth outcome. Descriptive statistics (*i.e.*, mean, standard deviation, frequencies and percent frequencies) were reported for all socio-demographic, behavior, and health characteristics. These variables were cross-tabulated by race, dietary patterns, and birth outcomes, and significant differences were assessed. Pearson correlations were conducted to assess linear relationship between birth outcomes and dietary patterns. Bivariate associations (least square means comparisons) were conducted between outcomes of interest (WLZ, WAZ, LAZ, HCZ) and maternal socio-demographic (age, length, race, education, health insurance) and health characteristics (BMI, tobacco use, gestational age, gravidity, total pregnancy weight gain, alcohol use, dietary patterns, use of multivitamin) and sex of the newborn.

Multivariable models for each outcome variable were constructed to describe their association with the exposure of interest (e.g., dietary patterns). These models were adjusted for any maternal socio-demographic or health characteristic that was independently and significantly associated with the outcomes of interest and with the exposure of interest in bivariate models. An alpha level of 0.05 was used for all statistical tests and *p*-values reported were not adjusted for multiplicity; therefore, the results must be considered in a hypothesis generating context. All analyses were performed using SAS version 9.3.

## 3. Results

### 3.1. Dietary Patterns and Nutrient Content

Table 1 describes each dietary pattern by its nutrient content and MyPyramid equivalents [46]. All of the dietary patterns were distinct in their macro and micronutrient contents. Below is a description of the most notable differences between patterns. The processed-Southern dietary pattern had the highest content in energy, total fat (% energy and total grams, including saturated, omega 3 fatty acids, monounsaturated, and polyunsaturated fats), total sugar, iron, zinc, sodium, and meats, and had the lowest content in whole grains. The processed dietary pattern was the highest *in* *trans* fats, total grains and potato servings. In contrast, the healthy-Southern dietary pattern had the highest content of fiber, folate, egg-meat equivalents, oils, vegetables (including dark green and orange vegetables, and tomatoes, excluding legumes and potatoes) and fruits (including fruit juice). The healthy-processed was characterized by high intake of nuts, seeds, whole grains, and dairy, as well as highly refined foods that are higher in simple sugars and fat. The healthy dietary pattern had the lowest energy, fat, total sugar, sodium, egg-meat equivalents and meats, and highest content of protein, carbohydrate, and soy legumes.

### 3.2. CANDLE Study Population Characteristics

Table 2 shows the socioeconomic characteristics of the CANDLE population sample for this study by race. There were significant differences in socio-demographic and behavioral characteristics of the racial groups. The African American mothers tended to be younger, have a higher body mass index (mean 28.8 kg/m^2^), be less likely to smoke, less likely to have completed higher education, and more likely to have Medicaid/Medicare insurance compared to the European Americans. Dietary patterns were significantly different by racial group, with European Americans and other race more likely to report a healthy dietary pattern. Mean birth weight for age, length for age, and head circumferences were significantly lower for African American offspring. European Americans were less likely to have more than one pregnancy.

### 3.3. Modeling

In bivariate analyses, eating processed and processed-Southern dietary patterns compared to healthy dietary pattern were negatively associated (*p* < 0.05) with weight-for-age Z-Score (WAZ), and head circumference Z-Score (HCZ), and positively associated with length-for-age Z-score (LAZ).

Variables that were associated with the various outcomes of interest in the bivariate analyses and also with the exposure of interest were included in the final multivariable model using race as a control variable. We also constructed each model for each race sub-group independently, controlling for potential confounders identified in the bivariate analyses. Since results by race groups were similar, we show the multivariable model that includes race as a control variable (Table 3), which is more powerful than the race-based analysis. For the outcome of HCZ, the healthy-processed dietary pattern was a positive significant predictor (HCZ β: 0.36; *p* = 0.019 compared to the healthy dietary pattern). None of the other dietary patterns were significant predictors of any birth size outcome after adjusting for confounders.

**Table 1:** Nutrient and MyPyramid values (per day) for dietary patterns in the Conditions Affecting Neurocognitive Development and Learning in Early Childhood (CANDLE) cohort. Values are means (SE).

Nutrient Values	Healthy (*n* = 135)	Healthy-Southern (*n* = 98)	Southern (*n* = 116)	Mixed (*n* = 440)	Healthy-Processed (*n* = 130)	Processed Southern (*n* = 136)	Processed (*n* = 99)	*p*-value
Energy (kcal)	1807 (38.6)	2319 (84.7)	1899 (62.1)	2337 (46.8)	2653.93 (72.31)	3051.51 (76.17)	2945.14 (85.79)	<0.0001
Fat (% energy)	34.3 (0.44)	35.8 (0.53)	36.3 (0.56)	36.3 (0.24)	36.36 (0.37)	37.97 (0.39)	37.66 (0.48)	<0.0001
Protein (% energy)	16.6 (0.20)	15.9 (0.26)	14.7 (0.21)	14.9 (0.12)	14.85 (0.17)	14.27 (0.19)	13.31 (0.24)	<0.0001
Carbohydrate (% energy)	52.02 (0.53)	50.71 (00.72)	50.95 (0.74)	50.64 (0.30)	50.73 (0.45)	49.01 (0.54)	50.46 (0.68)	0.0259
Total fat (g)	69.01 (1.78)	92.44 (3.7)	77.22 (2.91)	94.94 (2.06)	107.58 (3.18)	129.10 (3.60)	123.37 (3.95)	<0.0001
Omega 3 fatty acids (g)	1.67 (0.05)	2.36 (0.11)	1.66 (0.07)	2.05 (0.05)	2.28 (0.07)	2.39 (0.08)	2.19 (0.10)	<0.0001
Saturated fat (g)	21.78 (0.64)	28.63 (1.28)	25.61 (1.04)	31.15 (0.69)	35.21 ( 1.11)	42.86 (1.30)	41.32 (1.42)	<0.0001
Monounsaturated fat (g)	26.89 (0.72)	35.07 (1.4)	29.25 (1.13)	36.21 (0.80)	41.36 (1.24)	49.90 (1.39)	47.32 (1.51)	<0.0001
Polyunsaturated fat (g)	15.35 (0.44)	21.17 (0.87)	15.96 (0.60)	20.12 (0.46)	22.94 (0.70)	25.74 (0.73)	25.08 (0.85)	<0.0001
Trans fats (g)	2.22 (0.07)	2.82 (0.15)	2.84 (0.14)	3.67 (0.10)	4.41 (0.19)	5.15 (0.18)	5.36 (0.22)	<0.0001
Total sugar (g)	109.19 (2.97)	141.99 (6.74)	123.77 (5.49)	145.90 (3.26)	158.5 (5.60)	193.36 (6.75)	191.75 (8.46)	<0.0001
Fiber (g)	22.81 ( 0.73)	25.66 (0.99)	16.79 (0.64)	19.96 (0.42)	24.19 (0.83)	20.20 (0.62)	19.40 (0.65)	<0.0001
Fe (mg)	15.01 (0.44)	18.92 (0.77)	14.07 (0.47)	17.31 (0.38)	19.59 (0.60)	20.71 (0.56)	19.26 (0.66)	<0.0001
Zn (mg)	11.34 (0.33)	13.04 (0.48)	10.22(0.34)	12.91 (0.27)	14.38 (0.38)	15.88 (0.45)	14.77 (0.50)	<0.0001
Folate (ug)	352.91 (10.57)	439.75 (19.48)	250.90 (10.38)	306.41 (7.46)	359.08 (14.06)	279.95 (8.95)	251.20 (9.13)	<0.0001
Sodium (mg)	3058.62 (73.51)	4050.77 (157.30)	3190.14 (109.85)	3891.66 (85.42)	4393.10 (122.54)	5113.76 (136.66)	4636.01 (143.01)	<0.0001
Dairy-milk, cheese (1 cup equivalent)	1.90 (0.09)	1.79 (0.11)	1.35 (0.08)	1.84 (0.05)	2.06 (0.09)	1.73 (0.08)	1.68 (0.09)	<0.0001
Eggs-meat equivalent (1 egg = 1 oz)	0.35 (0.03)	0.92 (0.08)	0.71 (0.06)	0.61 (0.03)	0.47 (0.05)	0.89 (0.07)	0.61 (0.06)	<0.0001
Grain-total (1-oz equivalents)	5.52 (0.15)	6.67 (0.33)	5.41 (0.23)	7.00 (0.17)	8.45 (0.26)	8.49 (0.25)	8.6 (0.29)	<0.0001
Grain-whole (1-oz equivalent)	1.66 (0.07)	1.88 (0.12)	1.30 (0.09)	1.58 (0.05)	2.03 (0.09)	1.53 (0.08)	1.57 (0.10)	<0.0001
Legumes, soy (cup equivalent)	0.22 (0.03)	0.16 (0.03)	0.05 (0.01)	0.10 (0.01)	0.18 (0.04)	0.07 (0.01)	0.06 (0.01)	<0.0001
Meat-fish, chicken, meat (1 oz)	3.23 (0.13)	4.53 (0.27)	3.79 (0.17)	4.61 (0.13)	4.84 (0.19)	6.98 (0.27)	5.75 (0.28)	<0.0001
Nuts, seeds-(1-oz meat equivalent)	0.69 (0.06)	0.64 (0.07)	0.26 (0.04)	0.48 (0.03)	0.79 (0.05)	0.30 (0.03)	0.40 (0.05)	<0.0001
Beneficial Oils-dressings, fish, nuts, avocado (1 tsp)	2.66 (0.14)	3.06 (0.20)	1.71 (0.11)	2.48 (0.08)	3.00 (0.13)	2.37 (0.13)	2.42 (0.17)	<0.0001
Vegetables-dark green (cups)	0.62 (0.03)	0.81 (0.05)	0.37 (0.03)	0.45 (0.02)	0.50 (0.04)	0.29 (0.02)	0.23 (0.02)	<0.0001
Vegetables-not legumes/potatoes (cups)	1.92 (0.08)	2.39 (0.12)	1.24 (0.07)	1.50 (0.04)	1.75 (0.09)	1.25 (0.06)	1.10 (0.06)	<0.0001
Vegetables-orange (cups)	0.16 (0.01)	0.22 (0.02)	0.11 (0.01)	0.10 (0.004)	0.10 (0.01)	0.08 (0.01)	0.06 (0.01)	<0.0001
Vegetables-other, including tomatoes (cups)	1.14 (0.05)	1.36 (0.07)	0.74 (0.04)	0.94 (0.03)	1.13 (0.05)	0.84 (0.04)	0.76 (0.04)	<0.0001
Vegetables-potato (cups)	0.22 (0.01)	0.28 (0.03)	0.25 (0.02)	0.37 (0.01)	0.45 (0.03)	0.57 (0.03)	0.60 (0.03)	<0.0001
Fruit-total, including juice (cups)	1.68 (0.07)	2.55 (0.13)	1.82 (0.11)	1.68 (0.05)	1.65 (0.10)	1.78 (0.09)	1.43 (0.10)	<0.0001

**Table 2:** Population characteristics of mothers and newborns of the CANDLE study (*n* = 1151). Values are number (%) or means (standard deviation).

Characteristics	African American (*n* = 718)	European American (*n* = 401)	Other Race (*n* = 32)	*p*-value
*Maternal characteristics*				
Length, m	1.64 (0.07)	1.65 (0.07)	1.64 (0.06)	0.002
Age, years	25.13 (5.32)	28.65 (4.78)	30.00 (4.59)	<0.0001
Total weight gain, kg	14.78 (7.67)	14.83 (6.48)	13.41 (5.34)	0.80
Multivitamin, (% yes)	656 (91.4)	391 (97.5)	31 (96.9)	0.015
Body Mass Index, kg/m^2^	28.75 (8.07)	25.69 (6.02)	25.75 (5.58)	<0.0001
Tobacco, (% yes)	49 (6.8)	49 (12.2)	5 (15.6)	0.004
>1 pregnancy, (% yes)	512 (71.3)	254 (63.3)	23 (71.9)	0.021
*Education, n (%)*				<0.0001
<High school	489 (69.4)	123(30.7)	12 (37.5)	
>High school	219 (30.5)	278 (69.3)	20 (62.5)	
Insurance, *n* (%)				<0.0001
No insurance	15 (2.1)	4 (1.0)	2 (6.3)	
Medicaid/Medicare	511 (71.2)	86 (21.4)	9 (28.1)	
Private	192 (26.7)	311 (77.6)	21 (65.6)	
Alcohol use (% yes)	43 (6.0)	59 (14.7)	3 (9.4)	<0.0001
Premature delivery (% yes) *	68 (9.5)	26 (6.5)	2 (6.3)	0.18
*Diet Pattern, n (%)*				<0.0001
Healthy	7 (1.0)	121 (30.2)	7 (21.9)	
Healthy-Southern	74 (10.3)	13 (3.2)	11 (34.4)	
Southern	109 (15.2)	5 (1.2)	2 (6.3)	
Mixed	286 (39.8)	143 (35.7)	9 (28.1)	
Healthy-processed	30 (4.2)	98 (24.4)	2(6.3)	
Processed-Southern	131 (18.2)	3 (0.7)	1 (3.1)	
Processed	81 (11.3)	18 (4.5)	0 (0)	
*Newborn characteristics, mean (SD)*				
Weight-for-Length Z-score	−0.60 (1.23)	−0.57 (1.19)	−0.63 (0.98)	0.92
Weight-for-Age Z-score	−0.12 (0.91)	0.35 (0.92)	0.32 (0.91)	<0.0001
Length-for-Age Z-score	0.35 (1.18)	0.91 (1.23)	0.87 (1.27)	<0.0001
Head Circumference Z-score	−0.27 (1.22)	0.33 (1.23)	0.36 (1.22)	<0.0001

Significant differences across groups were tested using Chi-square or Kruskal Wallis. * Premature delivery defined as gestational age <37 weeks.

**Table 3:** Crude least square means (standard errors) and adjusted βeta estimates (standard errors) from generalized linear models of dietary patterns of mothers and newborn birth outcomes in Z-scores.

Dietary Patterns	Weight-for-Length Z-score (WLZ) (*n* = 923)	Weight-for-Age Z-score (WAZ) (*n* = 1011)	Length-for-Age Z-score (LAZ) (*n* = 1008)	Head Circumference Z-score (HCZ) (*n* = 999)
Healthy	Crude: −0.66 (0.10) Adjusted: Ref	0.33 (0.08) Ref	0.93 (0.12) Ref	0.19 (0.11) Ref
Healthy Processed	Crude: −0.49 (0.11) Adjusted: 0.16 (0.16)	0.36 (0.08) 0.12 (0.11)	0.89 (0.10) 0.07 (0.15)	0.45 (0.10) 0.36 (0.15) *
Healthy Southern	Crude: −0.76 (0.13) Adjusted: 0.17 (0.19)	−0.00 (0.09) * −0.09 (0.14)	0.60 (0.12) 0.05 (0.18)	−0.08 (0.13) 0.04 (0.18)
Mixed	Crude: −0.45 (0.06) Adjusted: 0.15 (0.14)	0.06 (0.05) * −0.01 (0.10)	0.47 (0.06) * −0.09 (0.14)	−0.02 (0.06) 0.09 (0.14)
Processed	Crude: −0.5 (0.15) Adjusted: 0.23 (0.19)	−0.05 (0.09) * −0.03 (0.14)	0.32 (0.13) * −0.17 (0.19)	−0.33 (0.14) * −0.18 (0.19)
Processed Southern	Crude: −0.74 (0.12) Adjusted: −0.07 (0.19)	−0.26 (0.08) * −0.15 (0.14)	0.30 (0.11) * −0.12 (0.18)	−0.39 (0.11) * −0.06 (0.19)
Southern	Crude: −0.89 (0.12) Adjusted: −0.28 (0.19)	−0.10 (0.09) * −0.07 (0.14)	0.59 (0.11) 0.17 (0.18)	−0.25 (0.12) * 0.05 (0.18)

This model was adjusted for age, race, pre-pregnancy BMI, education, alcohol and total weight gain. * *p* ≤ 0.05.

## 4. Discussion

This study examined the potential association between maternal dietary patterns during pregnancy and birth outcomes in a diverse population with historical high burden of low birth weight and other adverse birth outcomes [47,48,49]. The dietary patterns examined emerged from the foods that this population eats, and captured cultural food items related to traditional Southern cuisine, including fried fish, pig’s feet, tongue, pork, and dark green vegetables [40]. However, after controlling for confounders, our results do not offer strong evidence for the association between dietary patterns and birth outcomes in this population. Our findings indicate that only one dietary pattern (healthy-processed) characterized by intake of nuts, seeds, whole grains, and dairy, as well as highly refined foods that are higher in simple sugars and fat, is uniquely associated with higher HCZ compared to a healthy dietary patterns characterized by water, fruits and vegetables.

Our findings are somewhat comparable to those from previous publications in which dietary patterns characterized with nutrient-rich foods such as fruits and vegetables, whole grains, and water were associated with larger birth size outcomes[30,31,33,34,35,39]. The healthy-processed pattern was rich in whole grains, although one of the lowest in terms of dark green or orange vegetables. Our findings do not offer any evidence that either the “healthy” dietary pattern or the “processed” dietary pattern in this population was uniquely associated with birth weight or any other outcome, which is in contrast to what has been observed in other populations [30,31,34,35,39].

A potential explanation for this discrepancy is the antagonistic interaction among nutrients and food sources in the combined dietary patterns consumed by this population. For example, the healthy dietary pattern in the current study includes vegetables, fruits, non-fried fish and chicken, and water, similar to other studies, with the caveat that other studies have also included oils in their healthy pattern [16,17,33,34,50], contrary to the current study. In addition, the “healthy-Southern” pattern, but not the healthy pattern, is characterized by the highest intake of fruits, dark green and orange vegetables, fiber and folate. This division between healthy and “healthy-Southern” perhaps diluted the potential beneficial effects that a healthy diet may have had on birth outcomes. Similarly, the “healthy-processed” pattern, was characterized by high intake of nuts, seeds, dairy, whole grains, but also included processed and red meats, relatively high levels of saturated and trans fats, and refined sugars, which may have diluted some of the beneficial effects of the healthy foods on the other birth outcomes. Nuts, seeds and whole grains have high concentrations of unsaturated fats, protein, fiber, a variety of micronutrients and phytonutrients [51], and have been identified as part of a healthy diet pattern that was associated with favorable birth outcomes in other population studies [30,33,52]. The healthy-processed pattern was also characterized by dairy; dairy intake from milk and cheese can potentially provide optimum amounts of calcium and vitamin D. A few studies suggest that low calcium intake could have effects on the skeletal growth of the fetus, affecting birth, length and weight [53]. Optimum intake of calcium and vitamin D and low levels of parathyroid hormone are associated with decreased risk of SGA birth and a significantly higher birth weight, birth length, and head circumference [54]. These potentially antagonistic relationships could be assessed in a future study by nutrient-based patterns.

Although each dietary pattern has a variety of foods containing nutrients that have been shown to be antagonistic in their health effects, these dietary patterns were distinct in their nutrient profile and explained a large variance in food intake. Other potential explanations for our findings may have to do with socioeconomic or racial characteristics of the study population. Volgyi and colleagues [40] showed that women who were older and had higher level of education were more likely to eat a healthy dietary pattern than processed, Southern or mixed. Only seven African-American mothers consumed a healthy dietary pattern, whereas most European Americans consumed healthy or mixed patterns. Twenty-four percent of European American compared to only 4% of African American mothers consumed a healthy processed diet. Although our analyses statistically controlled for race, there may be contextual factors that affect birth weight and that are covariant with race, but are not completely captured by race.

The results of this study are limited by their reliance on self-reported dietary intake, the inherent limitations of quantifying dietary intake with a food frequency questionnaire, and the inevitable overlap between different dietary patterns. However, we took measures to overcome some of these limitations by excluding from the analyses all potential over and under-reporters of total caloric intake. In addition, the dietary patterns did show distinct factor loadings, suggesting that this population does eat diet patterns that combine “healthy” and “unhealthy” food items in an overlapping manner (*i.e.*, the mixed diet patterns).

## 5. Conclusions

In sum, our findings do not provide sufficient evidence that either the healthy or the processed dietary pattern in this population is uniquely associated with positive or negative birth outcomes. The mixed dietary patterns consumed by this study population may provide antagonistic relationships between foods and nutrients that result in null associations with birth outcomes. To further investigate this hypothesis, it would be necessary to discriminate the population by nutrient or micronutrient status, perhaps using biomarkers to potentially disentangle any antagonistic effects in foods or preparations. Our results also imply that there are other socio-environmental and maybe genetic aspects related to race in the Southern US that require careful further investigation in their association with birth outcomes.
